# IL-9 and IL-24 biomarkers in the transcriptional signature of contact dermatitis to methylisothiazolinone

**DOI:** 10.3389/fimmu.2025.1685396

**Published:** 2025-11-28

**Authors:** Taynah Cohen de Melo, Mirian Nacagami Sotto, Iara Grigoletto Fernandes, Emanuella Sarmento Alho De Sousa, Frederico Moraes Ferreira, Gabriel Benevides Simões, Yasmim Álefe Leuzzi Ramos, Marcella Soares Pincelli, Vitor Manuel Silva dos Reis, Maria Notomi Sato

**Affiliations:** 1 Laboratory of Medical Investigation 56, Department of Dermatology, University of São Paulo Medical School, São Paulo, Brazil; 2 Laboratory of Medical Investigation 50, Department of Pathology, University of São Paulo Medical School, São Paulo, Brazil; 3 Department of Immunology, Institute of Biomedical Sciences, University of São Paulo, São Paulo, Brazil; 4 Cellular, Genetic, and Molecular Nephrology Laboratory (LIM-29), Hospital das Clínicas, University of São Paulo Medical School, São Paulo, Brazil; 5 Department of Dermatology, University of São Paulo Medical School, São Paulo, Brazil

**Keywords:** allergic contact dermatitis, methylisothiazolinone (MI), RNA-Seq, differential gene expression, innate immune response, cytokines

## Abstract

**Introduction:**

Allergic contact dermatitis (ACD) is a cutaneous inflammatory disorder mediated by allergen-specific memory T cells. Methylisothiazolinone (MI), a preservative widely used in industrial and cosmetic products and a component of Kathon CG, has led to a substantial rise in ACD cases. Despite increasing sensitization rates, the innate immune mechanisms and transcriptional responses induced by MI in the skin remain poorly understood.

**Methods:**

Individuals with positive patch tests exclusively to MI were recruited at the Contact Dermatitis Clinic of Hospital das Clínicas (São Paulo). Participants were re-exposed to MI or saline, and skin biopsies were collected 48 hours later. Healthy MI-negative controls were also exposed to MI and saline. Histopathology and RNA-sequencing were performed. Differentially expressed genes (DEGs) were analyzed, and key findings were validated by qPCR and protein expression of IL-9 and IL-24.

**Results:**

Two distinct MI-responsive groups emerged among ACD patients:ACD-A (high responders): pronounced histopathology (spongiosis, microvesicles). ACD-B (low responders): milder reactions with absence of spongiosis. In ACD-A, MI exposure resulted in 1,588 upregulated and 2,090 downregulated genes compared to ACD-B. DEGs were enriched for innate immune and inflammatory pathways, including IL-24, IL-9, IL-13, and NTRK1 (upregulated), while IL-37 and IL-18 were downregulated. Compared to MI-negative ACD controls, ACD-A showed 1,169 upregulated and 321 downregulated genes. qPCR confirmed increased NTRK1 and IL-9 expression and reduced IL-18 levels. IL-9 and IL-24 protein levels were higher in the dermal layer of ACD-A.

**Discussion and Conclusion:**

MI-sensitized individuals exhibit heterogeneous innate immune responses despite uniformly positive patch tests. IL-9, IL-24, and NTRK1 appear to play important roles in the heightened inflammatory response observed in high-responder individuals, while downregulation of IL-18 and IL-37 may contribute to impaired regulatory pathways. These findings highlight previously undescribed heterogeneity in MI-induced ACD and identify potential targets for better understanding disease pathogenesis.

## Introduction

Allergic contact dermatitis (ACD) is a type IV hypersensitivity mediated by T cells, that affects 15–20% of the general population, regardless of age or gender (1). It is an adverse immunological reaction that occurs in individuals previously sensitized to an exogenous allergen, being influenced by factors such as the dose of the sensitizing agent, presence of irritants, changes in the epidermal barrier and genetic predisposition (2). ACD manifests after repeated contact with low molecular weight haptens or metal ions, manifesting with erythema, vesicles and, in chronic cases, lichenification and fissures (3). This condition significantly impacts the quality of life of patients and, particularly in occupational settings, imposes a substantial socioeconomic burden (4).

Although classically defined as a delayed-type hypersensitivity driven by adaptive immune cells, increasing evidence suggests that innate immune mechanisms play a crucial role in shaping and amplifying the T-cell response during both sensitization and elicitation phases of ACD (5). Innate sensors such as Toll-like receptors (TLRs) and inflammasome components can recognize danger-associated molecular patterns generated upon hapten exposure, leading to cytokine release (IL-1β, IL-18, TNF-α) and subsequent activation of adaptive responses (6). Investigating innate signaling cascades in MI-induced ACD may therefore reveal mechanisms that precede or amplify T-cell activation and identify novel therapeutic targets (7).

Methylisothiazolinone (MI), a widely used preservative due to its antimicrobial, fungicidal, and algicidal properties, is found in a variety of cosmetic, cleaning, and industrial products (8, 9). Previously, MI was marketed in combination with methylchloroisothiazolinone (MCI) in the Kathon^®^ CG formulation, at a ratio of 1:3 MI : MCI (10). This formulation was extensively used as a biocide in water-based paints, cleaning and polishing agents, laundry products, liquid detergents, and cosmetics since the 1980s (11). Its widespread exposure triggered outbreaks of ACD in Europe, becoming a significant cause of both occupational and non-occupational ACD (12–14). Although MCI was initially regarded the primary sensitizing agent, MI has since been recognized for its own potent allergenic capacity. This recognition prompted regulatory actions that restricted its concentration to 15 ppm in rinse-off cosmetic products (13, 15). Nevertheless, MCI/MI remains one of the most frequently used preservative systems in industrial and cosmetic products and, consequently, a common cause of ACD (16).

Our research group has been investigating the immunological mechanisms underlying ACD caused by MCI/MI. These investigations documented an upregulation of both regulatory components such as the cytokines IL-10 and TGF-β and the transcription factor Foxp3, and pro-inflammatory mediators, including IL-1β, IL-6, and TNF-β (7). Furthermore, we identified distinct perivascular infiltrates of M2 macrophages and Th2 lymphocytes in lesional skin, indicating a multifaceted immune response (17). Despite these advances, the specific immunological mechanisms driving ACD elicited exclusively by MI remain poorly understood.

Transcriptomic views may contribute in the identification of immune signaling pathways, differentially expressed genes (DEGs) and changes in cytokine expression involved in the immunopathogenesis of ACD associated with MI, in addition to supporting the development of more effective therapeutic and preventive strategies.

Our results reveal that the histopathological findings were consistent with the transcriptomic changes observed in the inflammatory pathway in response to MI. DEGs were barely present in the control groups, indicating an absence of inflammatory factors in individuals without a history of ACD. This was also verified in cases where the contact test was less intense. Furthermore, high MI responders exhibited upregulation of genes associated with IL-9, the IL-20 gene family, and the neurotrophic receptor tyrosine kinase (NTRK) gene in response to MI. These new factors in the ACD response could inform the development of more effective therapeutic and preventive strategies in response to MI.

## Material and methods

### Patient information

Individuals were selected at the Outpatient Clinic of the Dermatology Department of the Hospital das Clínicas da Faculdade de Medicina da USP (HC-FMUSP). The individuals were initially submitted to the Brazilian standard battery (Endoderme, São Paulo, SP, Brazil) containing 30 substances. Each allergen was placed in 8 mm Alergo Chamber containers (Neoflex, São Paulo, SP, Brazil) fixed to the back of the patients. Two readings of the test were carried out at 48 hours and 96 hours after its application. Of the total of 15 participants, 9 showed a positive patch test reaction to MI (++/+++), while the control group (HC-MI group; n = 6) consisted of individuals with negative patch test results for all tested substances and no history of atopic dermatitis, according to the criteria of Hanifin and Rajka (18). These individuals were recalled and submitted to a contact test with MI and saline, performing a skin biopsy after 48 hours of patch tests (**Table 1**).

**Table 1:** Demographic characteristics of individuals with methylisothiazolinone-associated allergic contact dermatitis (ACD) and healthy controls (HC).

Group*	Individuals	Sex	Age	Collection date	Duration of lesion	Occupation
A	ACD1	M	66	02/18/2022	10 Y	Retiree
	ACD3	F	59	04/26/2022	4 Y	Cleaning assistant
	ACD5	F	45	12/20/2021	6 M	Nursing assistant
	ACD6	F	55	07/30/2021	2 Y	Housewife
	ACD8	F	22	08/24/2022	5 Y	Student
Mean		1M/4F	~49.4	–	–	
B	ACD2	F	56	02/24/2022	3 Y	Housewife
	ACD4	M	69	11/11/2021	1 Y	Security
	ACD7	M	21	07/08/2022	6 M	Architect
	ACD9	F	42	05/24/2022	Not informed	Not informed
Mean		2M/2F	~47.0	–	–	
HC	6	4M/2F	~50.8	–	–	–

*They were divided into two groups, the more reactive group ACD-A MI and the less reactive group ACD-B MI, according to histopathological analysis. Y, years; M, months.

Exclusion criteria were immunosuppressants, history of autoimmune disease, melanoma, and seropositivity for HIV or hepatitis C or hepatitis B viruses. Also, use of systemic or topical corticosteroids less than 30 days before the test. All patients were informed about the research content, signing a free and informed consent form approved by the Ethics Committee for Analysis of Research Projects – CAPPesq of the Clinical Directorate of Hospital das Clínicas and the Faculty of Medicine of the University of São Paulo (72419617.4.0000.0068). All experimental protocols within this study were performed following the guidelines of the Ethics Committee of this institution and conformed to recognized standards of the Declaration of Helsinki.

### Obtaining skin biopsies

Skin fragments measuring approximately 6mm in diameter were obtained (through punch biopsies) from the region where the Alergo Chamber (Neoflex, São Paulo, SP, Brazil) was applied with MI or saline in individuals in the ACD and HC groups. Biopsies were performed 48 hours after the reapplication of patch tests. The tissue was collected after antisepsis with a 0.5% alcoholic chlorhexidine solution, placement of sterile cloth drapes, application of infiltrative local anesthesia, and injecting of 0.5mL of 2% lidocaine hydrochloride solution with norepinephrine hemitartrate 1:50,000. The region where the skin sample was removed was sutured using 3.0 black Nylon thread (Ethicon, Sommerville, NJ, USA). The collected tissue fragment was cut in half, in the vertical direction, with one of the halves stored in a 10% formaldehyde solution at room temperature for subsequent processing. The other half of the skin fragment was placed in an RNA later solution (Sigma Chemical Co, St. Louis, MO, USA) and kept at -80°C for further processing. At the end of the procedure, participants were instructed on local care and warned of the signs of possible complications. Two weeks after the biopsy, participants returned to have the stitches removed.

### Extraction and sequencing of total RNA from skin biopsies

To obtain mRNA, skin samples stored in RNA later were thawed and fragmented using the Tissue Ruptor apparatus (Qiagen, Hilden, Germany) and the RNeasy Plus Mini Kit (Qiagen) according to the manufacturer’s recommendations. The integrity, quality, and quantification of the samples were verified using the Tapestation 4200 apparatus (RNA ScreenTape, Agilent, Santa Clara, CA, USA). Libraries for sequencing were constructed with purified RNAs and the TruSeq Stranded mRNA Library Prep kits (Illumina, San Diego, CA, USA), following the manufacturer’s instructions. These libraries were sequenced on the NextSeq 500 platform (Illumina) using the V2.5 Mid Output kit, 2 x 75 cycles. Samples quality checking and sequencing were performed at the Large-Scale Sequencing Laboratory of FMUSP (Premium Network).

Quality of the raw FASTQ files was initially assessed using FastQC v0.11.9. Fastp v).23.2 was employed to remove the sequencing adapters and low-quality bases using default parameters (19). The high-quality reads were then aligned to the GRCm38 reference genome assembly version 104 using the Subread package v2.0.3 (20). The gene-level count matrix was generated using featureCounts from the same Subread package version (21).

Differential gene expression (DEGs) analysis was conducted using the R package DESeq2 v3.20 (20). Prior to analysis, genes with minimal expression, defined as having a cumulative raw count of one or less across all samples, were removed. Comparisons were performed to identify DEG between the following ACD-A MI, ACD-A Sal, ACD-B MI, ACD-B Sal and HC MI and the healthy control group. Statistical significance was determined using the Wald test, and the resulting p-values were adjusted for multiple comparisons via the Benjamini-Hochberg procedure. A gene was considered differentially expressed at an adjusted p-value (padj​) of ≤ 0.05. No explicit log2​(fold change) threshold was applied for DEG selection.

To interpret the biological significance of the DEG from each comparison, a functional enrichment analysis was performed using the fGSEA R package (22). The analysis encompassed the molecular pathways databases KEGG (22), Reactome (23) and Wikipathways (24). Pathways were considered significantly enriched at a Benjamini-Hochberg adjusted p-value of ≤ 0.05.

The RNA sequencing data presented in this study have been deposited in GEO under accession code GSE305246.

### Immunohistochemistry

The skin biopsy fragments stored in formalin were processed to generate histological sections 4-μm thick. The slides were then deparaffinized and incubated with 3% hydrogen peroxide and washed. The slides were incubated with 10% milk, washed and incubated with monoclonal of rabbit anti-human IL-9 antibodies (Abclonal) and Il-24 (Invitrogen) for 18 hours at 4°C. The following day, the slides were washed with PBS, incubated with Novolink post-primary (Leica Biosystems), and then incubated with DAB chromogen solution, washed, and counterstained with hematoxylin. For analysis and quantification, stained slides were scanned and digitized at 20X magnification using the Aperio ScanScope XT imaging system (Aperio, Vista, CA) and visualized using ImageScope software. Subsequently, the samples were analyzed using the Image-Pro Plus software (Media Cybernetics). For the analysis, the epidermal region was delineated to calculate the proportion of the stained area and the staining intensity relative to both the total epidermal area and the length of the basement membrane. In the dermis, the stained area and staining intensity were quantified in relation to the total dermal area. The resulting data were then processed using GraphPad Prism 9 software (GraphPad Holdings, San Diego, CA, USA) for graphical representation.

### Gene expression by real-time PCR

The expression of upregulated targets according to RNAseq was confirmed by real-time PCR in skin samples from the ACD MI and HC MI groups. The RNA extracted from the biopsies was quantified in a Nanodrop spectrophotometer (ThermoScientific, USA). Subsequently, the reverse transcriptase reaction with the iScript kit (Biorad) converted the RNA into cDNA. To perform the real-time amplification reaction, 5 μL of cDNA (20ng/mL), 7 μL of SYBR^®^ Green solution (Applied Biosystem), and 2 μL (1 mM) of the primers of the target genes and the reference gene (GAPDH), which Invitrogen synthesized, were used for each sample (**Supplementary Table 1**). The PCR reaction was performed on the 7500 platform (Applied Biosystems), and the results were analyzed using the 7500 Software v2.0.6 program (Applied Biosystems), according to the delta-CT method (25). The sequence of the oligonucleotides used in the PCR reaction is described in **Supplementary Table 1**.

### Statistical analysis

Comparisons were performed according to the *Mann-Whitney* test. The significance level considered was *P* ≤ 0.05. All statistical and graphical representations were performed with GraphPad Prism 9 software (GraphPad Holdings, San Diego, CA, USA).

## Results

### Histopathological analysis of MI-induced ACD

**Figure 1:**
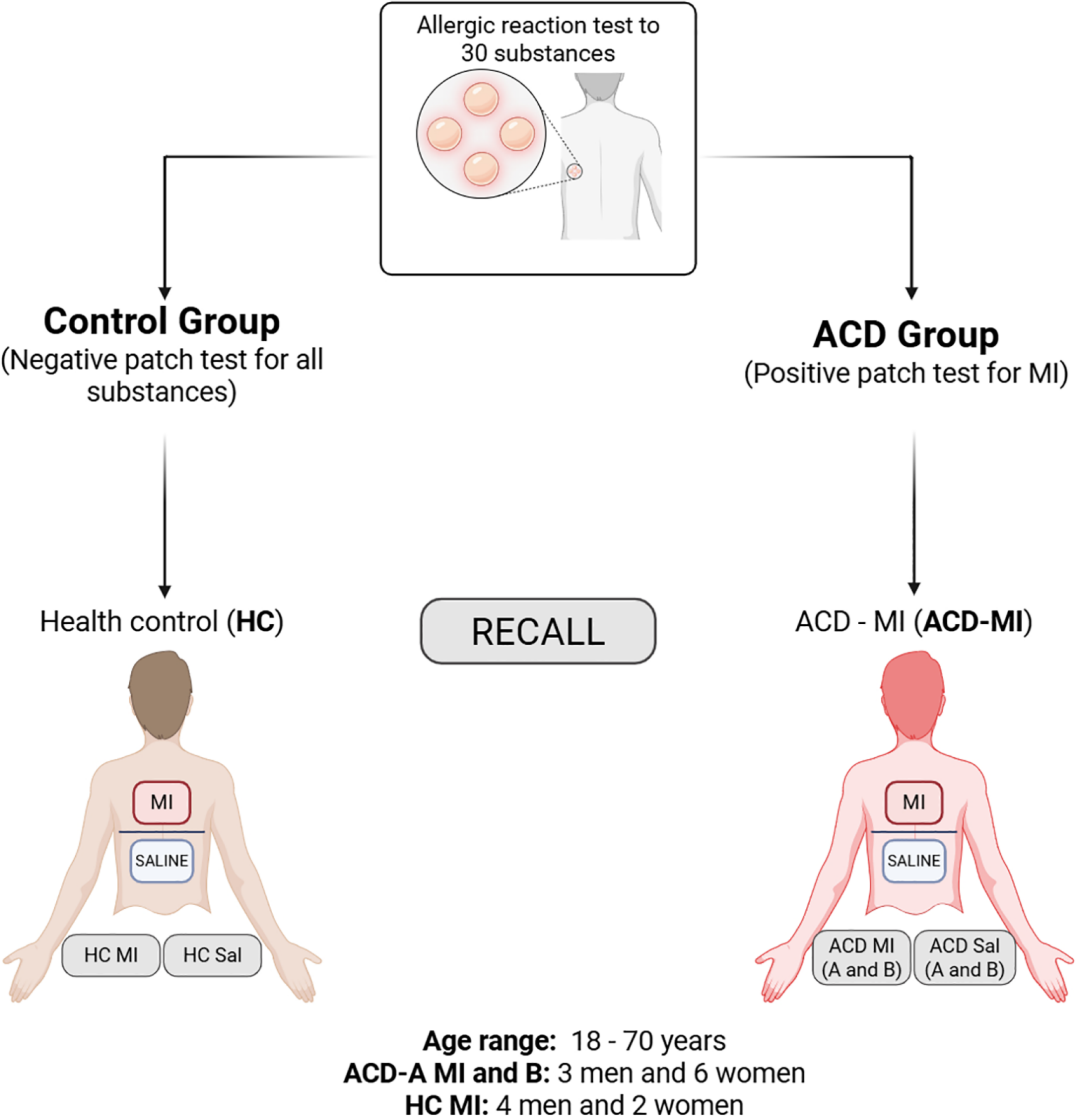
Experimental design overview. Schematic representation of the study design for patch testing and recall response to methylisothiazolinone (MI). The control group (HC) included individuals with negative patch tests for all substances, while the allergic contact dermatitis group (ACD-MI) comprised individuals with positive patch tests for MI. Both groups underwent patch application with MI and saline (Sal) for subsequent comparative analyses. Age range: 18–70 years. ACD MI-A and MI-B: 3 men and 6 women; HC MI: 4 men and 2 women. Created in BioRender. Sousa, D. (2025) https://BioRender.com/pu87oh0.

The study cohort included 9 participants with a clinical diagnosis of ACD and a positive patch test reaction (++ or +++) exclusively to methylisothiazolinone (MI). An additional six individuals with negative patch tests and no history of atopic dermatitis served as the healthy control group (HC) (**Figure 1**).

**Figure 2:**
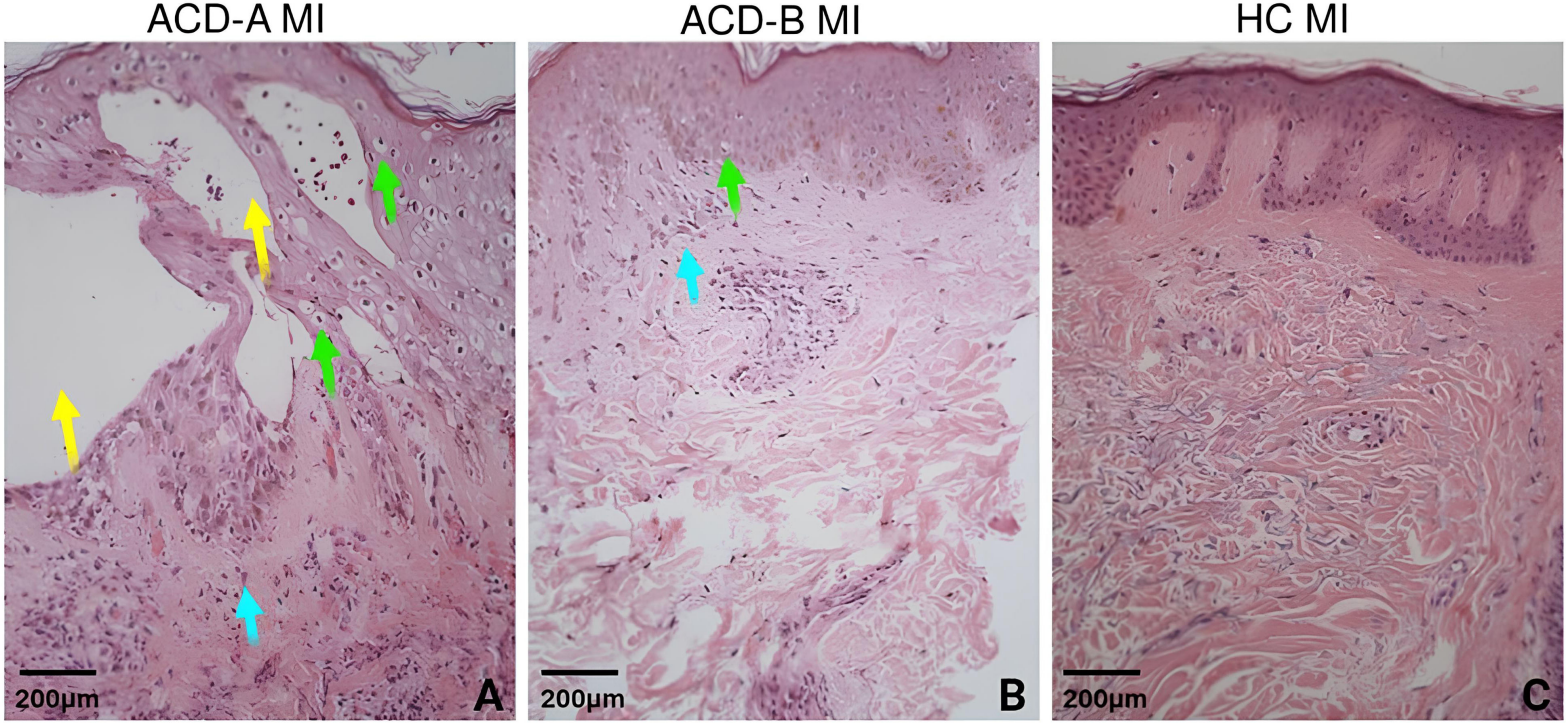
Histopathological overview. Representative histological images of skin biopsies stained with hematoxylin and eosin (H&E), illustrating key features of allergic contact dermatitis (ACD). Findings include the presence of microvesicles (yellow arrows, panel A), spongiosis (green arrows, panels A and B), and lymphocytic exocytosis (blue arrow, panel A and B). **(A)** Representative histological image showing an intense response to methylisothiazolinone (MI). **(B)** Representative histological image showing a mild/moderate response to MI. **(C)** Representative histological image from the control group exposed to MI.

Histological analysis revealed characteristics differences correlated with the intensity of the patch test to MI (**Table 2**). Five samples (cases 1, 3, 5, 6, and 8) exhibited marked histopathological changes, with scores of +3 or +4. These alterations included intense spongiosis, prominent micro vesicle formation (3+ to 4+), vacuolar and reticular degeneration (up to 3+), along with dense lymph histiocytic infiltrates containing neutrophils and eosinophils—particularly evident in case 8, which presented 4+ LHN with NeEo in both superficial and interstitial dermal compartments. Additionally, significant edema was observed (e.g., cases 1, 3, 5, and 8 with edema scores of 2+) (**Figure 2A**, **Table 2**). Based on histopathological findings, the samples with strong clinical reactivity (3+) were classified as the ACD-A MI group.

**Table 2:** Histopathological features of the epidermis and dermis in nine individuals with methylisothiazolinone-associated allergic contact dermatitis (MI-ACD).

Case	Epidermis					Dermis			
	Spongiosis	Microvesicles	Vacuolar change	Reticular change	Lymphocytic exocytosis	Edema	Superficial perivascular infiltrate	Interstice	Endothelial swelling (edema)
1^+^	3+	4+	3+	2+	2+	1+	3+ LHN	3+LHNe	2+
2	2+	0	2+	0	2+	0	2+ LHEo	1+ LH	1+
3^+^	3+	4+	3+	2+	2+	1+	4+ LHEo	3+ NeEo	2+
4	0	0	0	0	0	0	1+LHEo	0	0
5^+^	4+	3+	2+	2+	3+	1+	3+ LH	3+ LHEo	2+
6^+^	3+	2+	2+	1+	2+	1+	3+ LH	3+ LHEo	1+
7	2+	1+	2+	0	2+	1+	2+ LHEo	2+ NeEo	1+
8^+^	2+	2+	3+	0	3+	1+	4+ LHNeEo	4+ LHEo	2+
9	0	0	0	0	0	0	2+ LH	0	0

Histopathological alterations were graded as follows: 0 = absent; 1 = weak; 2 = moderate; 3 = high/intense; 4 = strong. Inflammatory cell infiltrates are denoted as: LH, lymphohistiocytic infiltrate; LHN, lymphohistiocytic infiltrate with neutrophils; LHEo, lymphohistiocytic infiltrate with eosinophils; LHNeEo, lymphohistiocytic infiltrate with neutrophils and eosinophils. “+ number” indicates the cases within group A.

In contrast, cases 2, 4, 7, and 9 showed milder histopathological alterations, with scores ranging from +1 to +2, characterized by moderate spongiosis, absent or minimal micro vesicle formation (0 to 1+), and mild dermal inflammatory infiltrates (maximum 2+ LH or LHEo) with little to no edema (**Figure 2B**; **Table 2**). These individuals were assigned as ACD-B MI group.

All control cases—including MI-positive ACD samples treated with saline and MI-treated samples from MI-negative individuals—showed no relevant histopathological alterations in either the epidermis or dermis (as shown in **Figure 2C**).

Altogether, these findings demonstrate that MI elicits a heterogeneous histopathological response, allowing for the stratification of ACD patients into high-responder (ACD-A) and moderate-responder (ACD-B) subgroups based on the severity of tissue inflammation and damage.

### Altered gene expression in ACD to MI

Based on the histopathological findings, we next evaluated the molecular profile of these groups by performing RNA sequencing on patch test samples from all groups. Based on 9 patients, it was divided into high responders (ACD-A MI, n = 5) and those with a moderate response (ACD-B MI, n = 4). Basal reactivity to saline was also assessed in each group. In addition, individuals with negative patch tests, referred to as healthy controls (HC, n = 6), were also exposed to both MI and saline.

**Figure 3:**
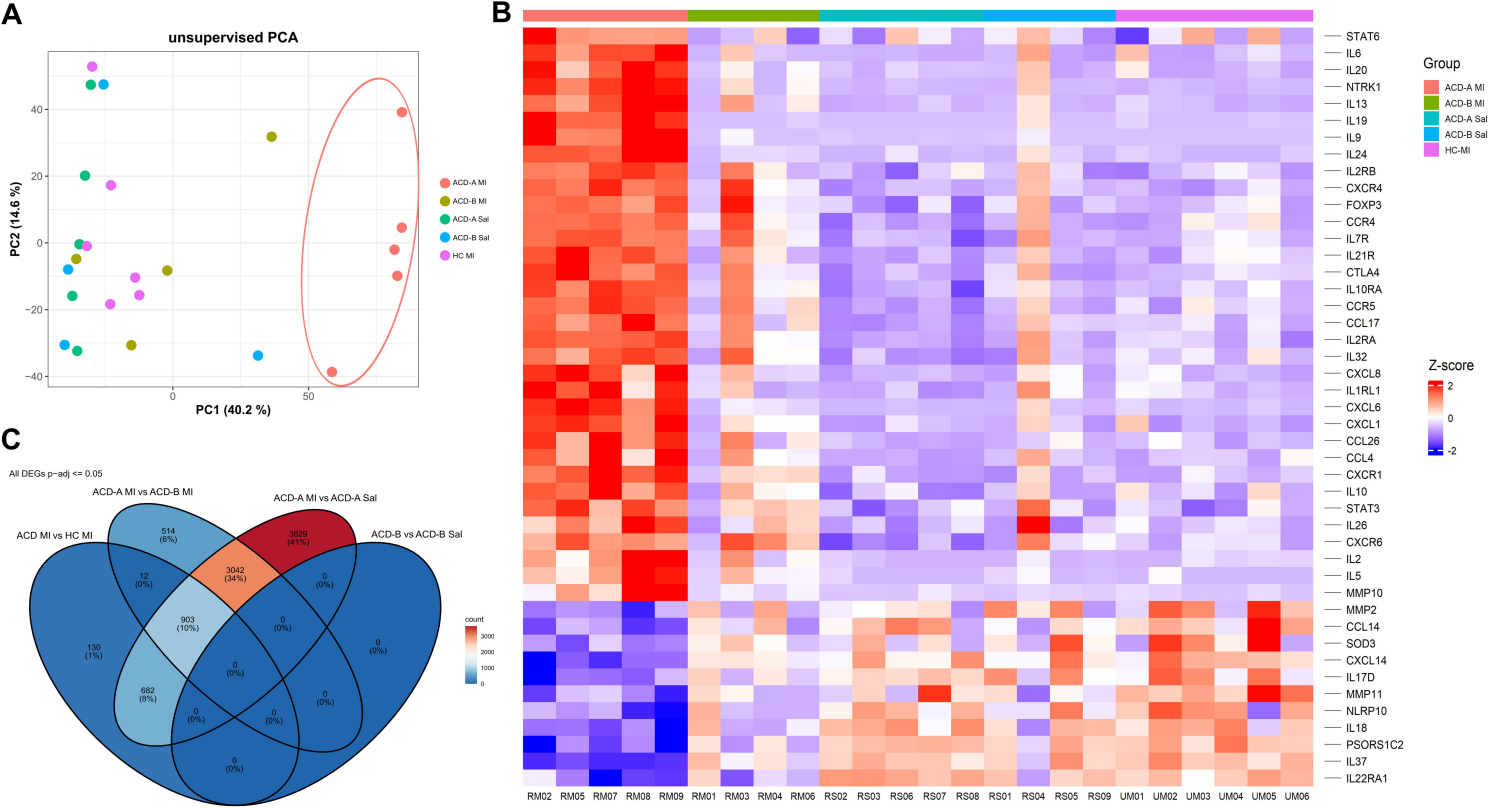
Transcriptomic Profiling of Skin Samples. **(A)** Principal Component Analysis (PCA): The PCA plot demonstrates the distinct clustering of transcriptomic profiles, with ACD-A MI samples (red) forming a separate group, indicating a unique transcriptional signature compared to other conditions. **(B)** Supervised Heatmap: The heatmap shows the expression patterns of differentially expressed genes (DEGs) across all experimental groups, highlighting upregulation (red) and downregulation (blue) of key inflammatory and immune-related genes in ACD-A MI. **(C)** Venn Diagram: The Venn diagram illustrates the overlap of significantly differentially expressed genes (adjusted p-value ≤ 0.05) among the experimental comparisons, emphasizing a substantial number of unique DEGs in the ACD-A MI group.

Principal Component Analysis (PCA) of the transcriptomic data demonstrated a clear segregation of the experimental groups based on their overall gene expression profiles (**Figure 3A**). The first two principal components PC1 and PC2 accounted for a combined 54.8% of the total explained variance (40.2% and 14.6%, respectively). Notably, the ACD-A MI group formed a distinct and tight cluster, separating completely from the ACD-B MI and control groups, which clustered together, indicating a unique and robust transcriptional response in high responders.

Consistent with the PCA, hierarchical clustering of the differential gene expression (DEG) revealed a distinct expression profile between ACD-A MI and the other groups (**Figure 3B**). Notably, genes such as IL-19, CXCL1, CXCL8 and NTRK1 were highly expressed in this group but downregulated in the others. Conversely, genes such as the anti-oxidant enzyme SOD3 (anti-oxidant enzyme), the inflammatory regulator NLRP10, PSORS1C2 exhibited reduced expression exclusively in the ACD-A MI group, while being highly expressed in the others.

To identify shared and unique transcriptional signatures, we compared the DEG across all groups (**Figure 3C**). A total of 3,042 DEGs (34%) were commonly found in two comparisons (ACD-A MI *vs*. ACD-A Saline and ACD-A MI *vs*. ACD-B MI) while 3,629 DEGs (41%) were uniquely detected in the ACD-A MI vs. ACD-A Sal group, indicating a distinct transcriptional response in MI-sensitized individuals. Additionally, exclusive DEGs were observed in ACD-A MI vs. ACD-B MI (514 genes; 6%) and ACD-A MI vs. HC MI (130 genes; 1%), indicating specific molecular signatures related to group differences. No DEGs were identified in the ACD-B MI vs. ACD-B Sal comparison (0%), showing a minimal response in this group. Positive groups to MI showed that ACD A carried 514 genes more than ACD B. These findings highlight the robust gene expression changes specifically associated with MI sensitization in ACD-A group.

**Figure 4:**
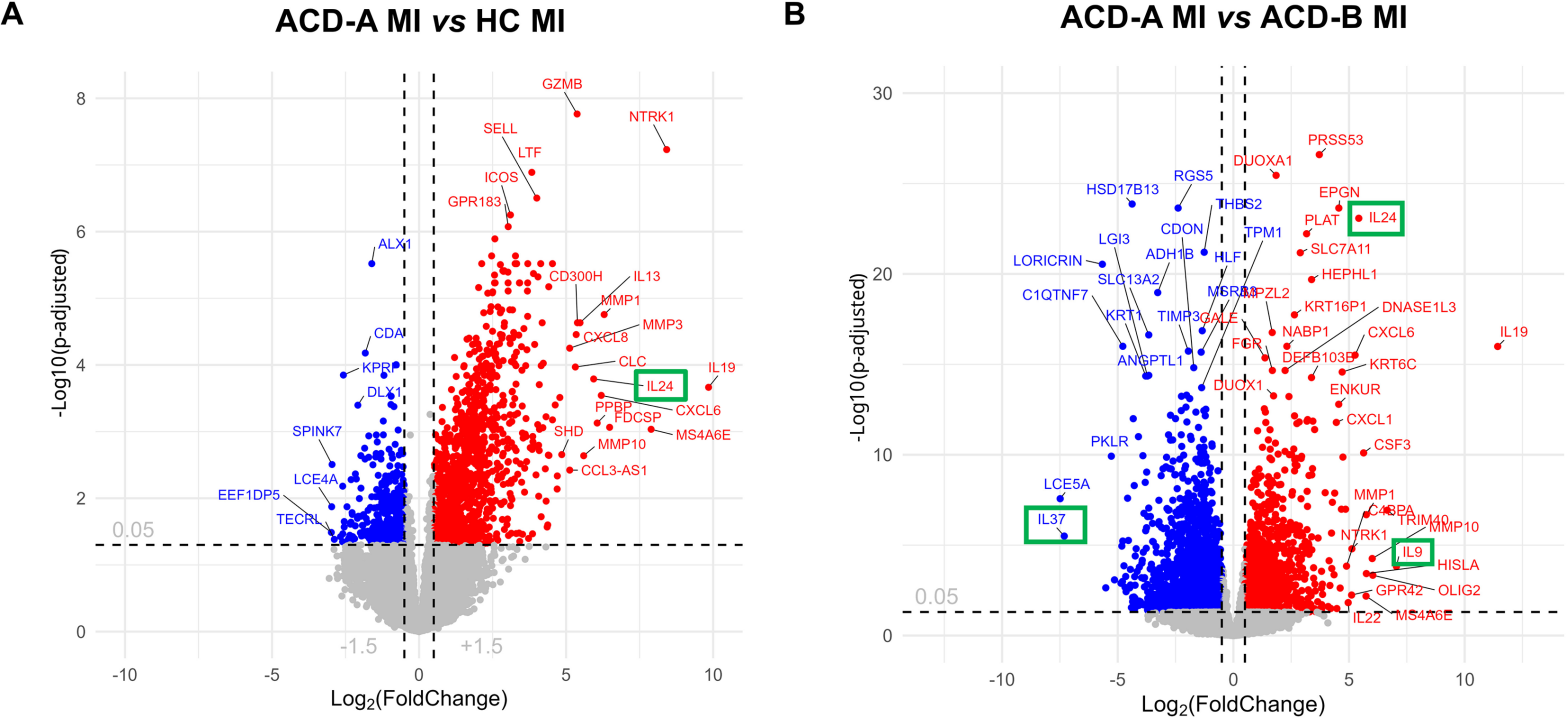
Volcano plots showing differentially expressed genes (DEGs) between study groups. **(A)** Comparison between ACD-A MI and HC MI groups, with 1,160 genes upregulated, 321 downregulated, and 15,777 not significant. **(B)** Comparison between ACD-A MI and ACD-B MI groups, with 1,588 genes upregulated, 2,090 downregulated, and 13,409 not significant. Genes significantly upregulated are shown in red, downregulated in blue, and non-significant genes in grey. The x-axis represents the log_2_ fold change (log_2_FC), and the y-axis represents the –log_10_ adjusted p-value.

The volcano plot analysis further details marked transcriptional differences between ACD-A MI and HC MI groups, with a total of 17,258 genes, being 1,160 genes upregulated, while 321 were downregulated (**Figure 4A**). Among the upregulated genes, IL-24, IL-19, IL-13, NTRK1, and CXCL6 stood out, evidencing the activation of inflammatory pathways, cytokine signaling, neuronal growth factors, and chemokines.

A comparison of ACD-A MI (ACD-A) with ACD-A upon saline application (ACD-A sal) it was detected 3,186 genes were upregulated, including NTRK1, IL-6, IL-13, and CXCL6, consistent with previous findings, and also IL-9 (**Supplementary Figure 1**). It is remarkable that there are more upregulated DEGs in this condition despite there are some common DEGs in the comparison with the ACD-negative individual. Notably, IL-37 and FLG2, associated with anti-inflammatory regulation and skin barrier function, respectively, were downregulated. This data shows an impaired barrier function and decreased genes with regulatory function favoring the amplification of inflammation in ACD-A MI patients. We can suggest that comparing the ACD-A MI individual with saline (ACD A-saline) is interesting because it shows which symptoms are induced by the allergen. More so than comparing with a healthy individual.

Comparison of the more reactive ACD-A MI group with the less reactive ACD-B MI, (**Figure 4B**), revealed ACD-A MI exhibiting a higher expression of inflammatory genes and immunological factors pathways, including CXCL1, IL-19, IL-24, and IL-9. In contrast, there was downregulation of genes related to the integrity of the skin barrier and the anti-inflammatory response, such as LORICRIN and IL-37. Curiously, similar DEGS were detected in the comparison of ACD-A MI with ACD-A saline (**Supplementary Figure 1**), such as up IL9 and down of IL-37.

The clear distinction in expression profiles observed in the heatmap and the volcano plots prompted a more detailed investigation of the enriched pathways, intending to understand the biological processes underlying the differences between the groups.

**Figure 5:**
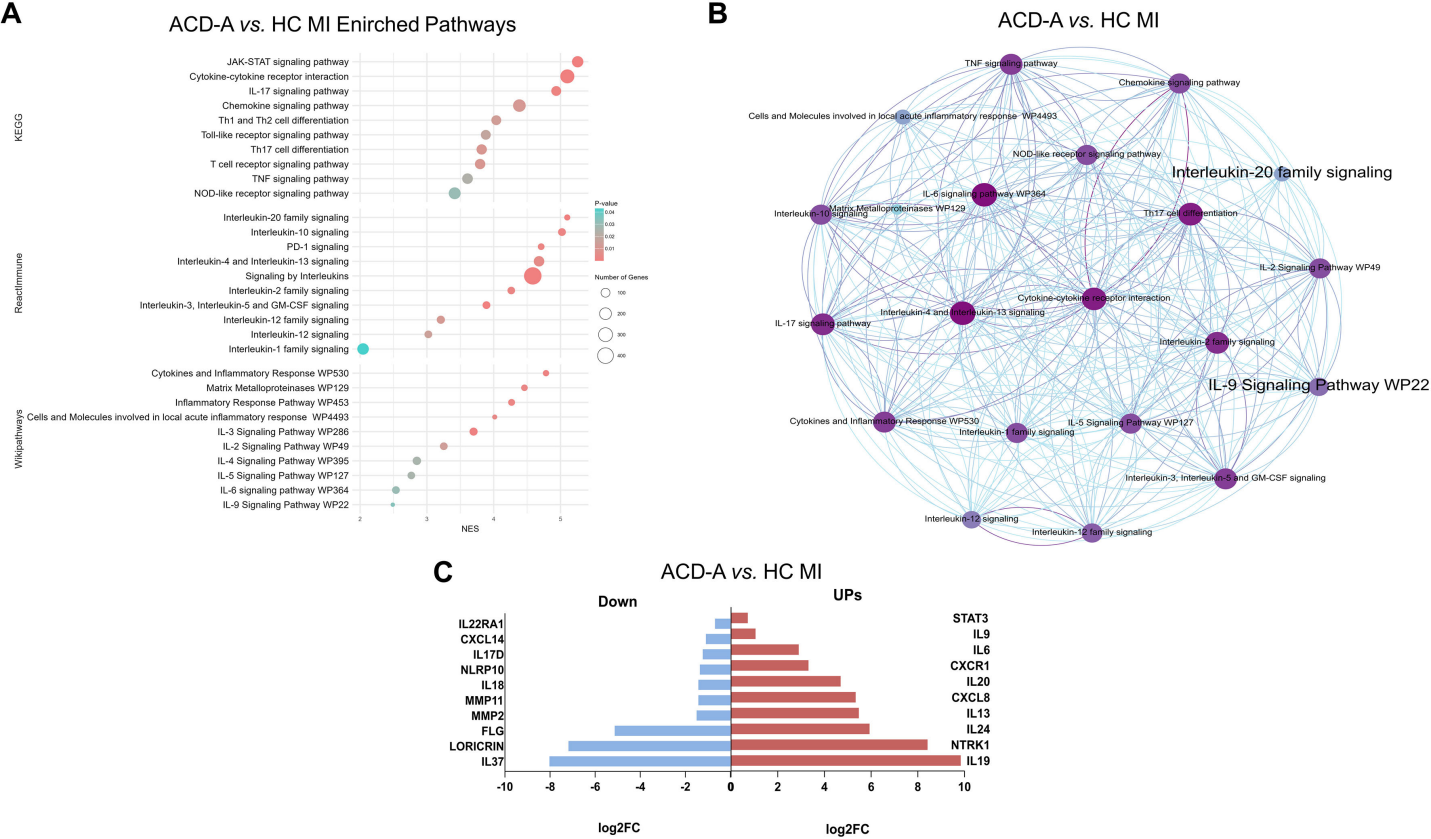
Enriched signaling pathways in ACD-A MI compared with HC MI. **(A)** Bubble plot showing enriched signaling pathways identified by KEGG, Reactome, and WikiPathways analyses. The x-axis represents the normalized enrichment score (NES), bubble size indicates the number of genes, and bubble color represents the adjusted p-value. Prominent pathways include IL-6 signaling, IL-4/IL-13 signaling, IL-17 signaling, TNF signaling, Th17 cell differentiation, chemokine signaling, and cytokine–cytokine receptor interaction. **(B)** Network representation illustrating the interconnections among enriched pathways, highlighting a complex immune network dominated by cytokine-driven inflammatory signaling cascades. **(C)** Bar plot showing the distribution of upregulated (red) and downregulated (blue) genes across the enriched pathways. These findings indicate a strong Th2/Th17-mediated inflammatory profile in ACD-A MI, with contributions from both adaptive and innate immune responses.

Analysis of immune factors and signaling pathways using the KEGG, Reactimmune, and WikiPathways databases revealed several enriched pathways in the comparison between the ACD-A MI group and the HC MI group. These included interleukins signaling, cytokine–cytokine receptor interaction, Jak–STAT, chemokine signaling, and pathways involving the IL-20 family, IL-4/IL-13, IL-17, IL-9, among others (**Figure 5A**). The connections between these factors are illustrated in **Figure 5B**. In the ACD-A MI group, analysis of the top ten DEGs highlighted an inflammatory signature, with overexpression of genes such as IL9, NTRK1, IL13, IL19, and CXCL8, which are known to mediate Th2- and Th9-driven immune responses, epithelial activation, and chemotaxis (**Figure 5C**). Conversely, downregulated genes were associated with epidermal barrier function (e.g., LORICRIN [LOR], FILAGGRIN [FLG]) and anti-inflammatory regulation (e.g., IL37, NLRP10, and IL18 (**Figure 5C**)). These findings support the notion that ACD lesions lead to a disrupted skin barrier and a skewed immune response, contributing to the allergic and inflammatory phenotype.

### DEGS validations in response to MI

Based on the RNA-seq results, we validated some selected DEGs such as NTRK1, IL-9, IL-6, IL-13, IL-18 and CXCL8/IL-8 by qPCR technique utilizing biopsy samples from the patch test.

**Figure 6:**
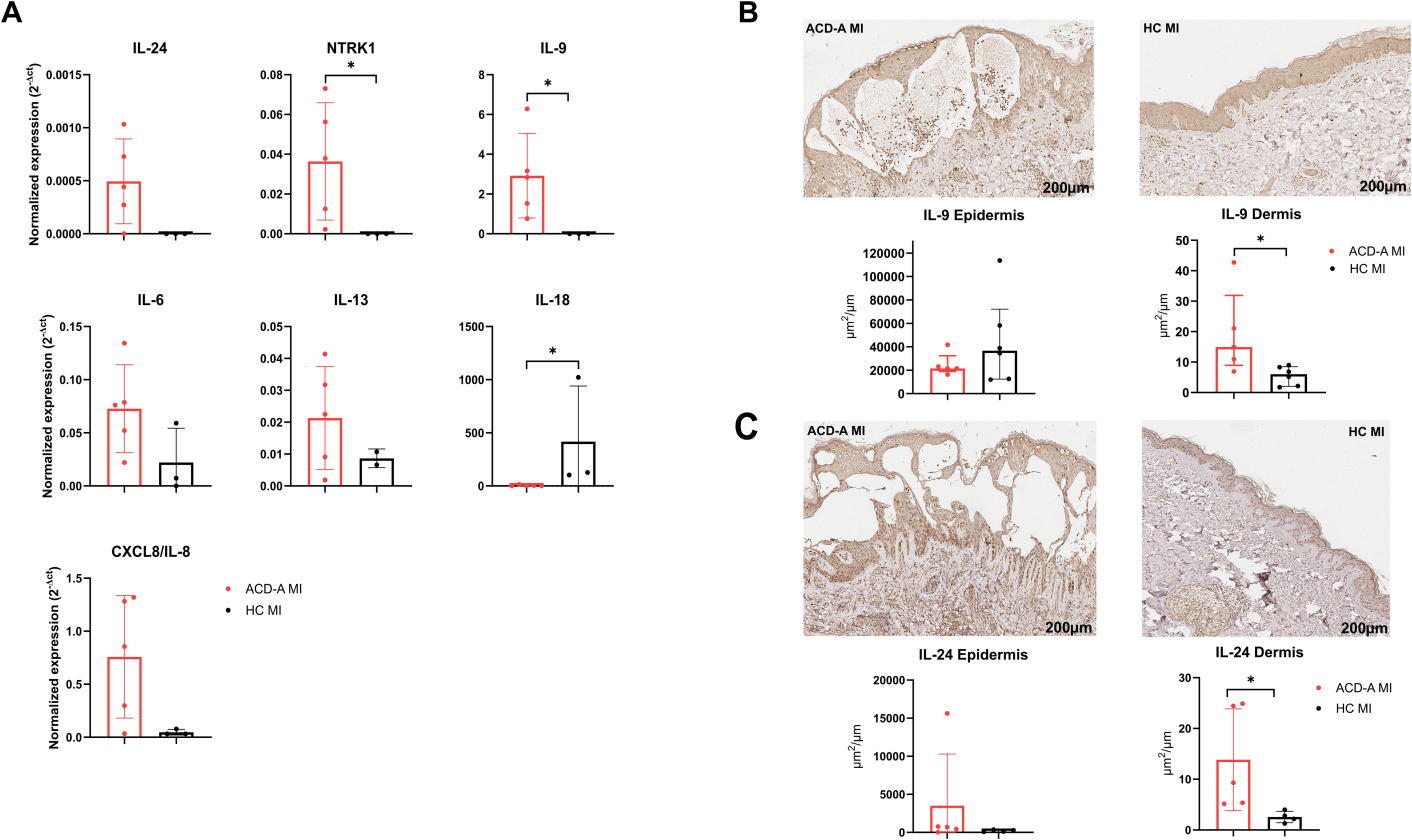
mRNA and protein expression of selected cytokines and receptors in ACD-A MI and HC MI groups. **(A)** Quantitative PCR analysis showing increased mRNA expression of NTRK1, IL9 and reduced IL18 in ACD-A MI compared with HC MI controls. Data are presented as normalized expression (2^-ΔCt) values. **(B)** Immunohistochemical staining for IL-9 in skin biopsies from an ACD-A MI patient (red bar, showing markedly higher staining intensity) and an HC MI control (black bar). Quantification reveals increased IL-9 staining in both the epidermis and dermis, with a more pronounced increase in the dermis. **(C)** Immunohistochemical staining for IL-24 in skin biopsies from an ACD-A MI patient (black bar) and an HC MI control (red bar). Quantification shows elevated IL-24 staining in the epidermis and dermis of ACD-A MI compared with HC MI. Scale bars = 200 μm. These findings demonstrate that IL-9 and IL-24 are upregulated at both the transcriptional and protein levels in ACD-A MI, particularly within the dermis, suggesting their potential contribution to the Th9/IL24-driven inflammatory response in MI-induced allergic contact dermatitis. The symbol “*” indicates statistical significance (p < 0.05).

Gene expression revealed upregulation of NTRK1 and IL9 in the ACD-A MI group compared to the HC MI controls (**Figure 6A**). NTRK1, a key receptor involved in neurogenic inflammation, and IL9, a cytokine associated with Th9-mediated immune responses, may contribute to the inflammatory mechanisms observed in MI-induced ACD.

In contrast, expression of IL-18 was lower in the ACD-A MI group than in the HC MI group, despite the heterogeneity of the samples. Moreover, we can observe that ACD-B MI has similar profile with healthy control group (**Supplementary Figure 2**).

The findings are consistent with the RNA-seq results, reinforcing the importance of targeted cytokine signaling in the pathophysiology of ACD-A MI One limitation of our study is the small number of samples analyzed, but we have to consider that each individual was submitted to two biopsies.

We chose IL-9 and IL-24 for further validation because it is one of the main candidates involved in the inflammatory response characteristic of ACD-A MI. Protein expression was evaluated by immunohistochemistry (IHC) to quantify IL-9 in lesional skin samples, allowing for assessment in both epidermal and dermal compartments (**Figure 6B**). We observed increased IL-9 expression in the dermis of the ACD-A MI group compared to controls. Moreover, the dermal IL-24 expression was also increased ACD-A MI group (**Figure 6C**).

Epidermal analysis was partially compromised due to the presence of spongiosis and intraepidermal vesicle formation, which limited accurate measurement by the software.

These findings show intense IL-9 and IL24 dermic expression in the response to MI in ACD-A group.

### CD4^+^ T cell dynamics in ACD-A MI

**Figure 7:**
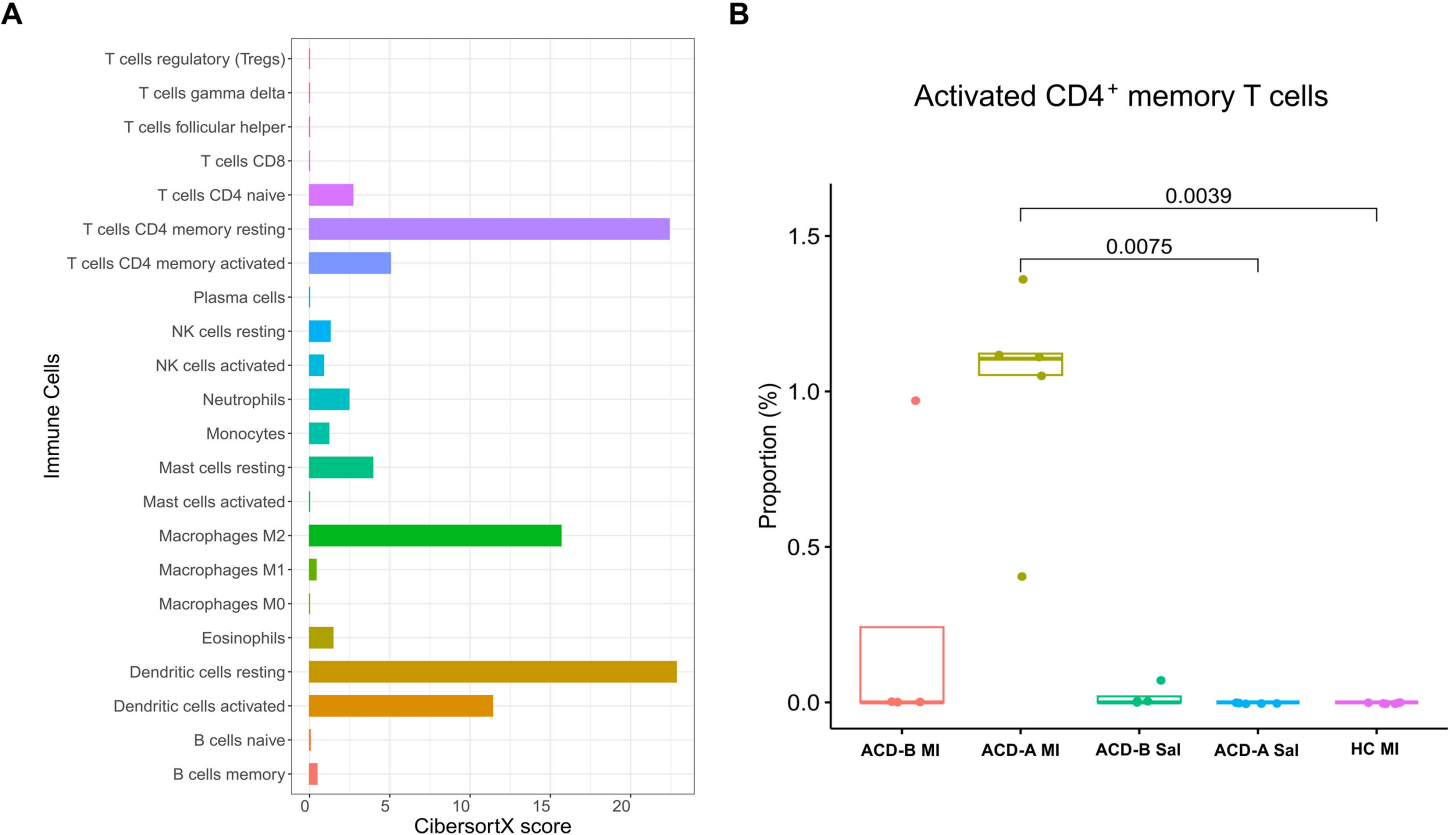
Immune cell composition in ACD-A MI, ACD-B MI, and control groups. **(A)** Relative abundance of immune cell subsets estimated using the CIBERSORTx algorithm. Higher proportions of resting CD4^+^ memory T cells, M2 macrophages, and resting dendritic cells were observed in ACD-A MI samples. **(B)** Box plot showing the proportion of activated CD4^+^ T cells across study groups. ACD-A MI exhibited significantly higher proportions compared with its own saline control (*p* = 0.0075) and the healthy control MI group (*p* = 0.0039).

To characterize the immune cell composition within the lesional skin of ACD patients, we performed deconvolution analysis using CIBERSORTx (**Figure 7A**). This approach revealed an enrichment of resting CD4^+^ memory T cells as the predominant T cell population across all groups. Additionally, a marked presence of M2 macrophages and resting DCs were observed. An increased proportion of activated CD4^+^ memory T cells were detected in the ACD-A MI group compared to the other cohorts (**Figure 7B**). This analysis shows a participation of memory T cell activation in ACD in group A when re-challenged with MI.

## Discussion

Our results revealed a molecular transcriptional profile in the ACD response to MI that was consistent with the histopathological findings of positive patch tests. Notably, among individuals with positive patch tests, a subset (group A) exhibited a robust DEG signature associated with inflammatory pathways, cytokine signaling, and neuroimmune responses, which correlated with more severe histopathological alterations, as supported by the unsupervised PCA and DEG heatmap analyses. In contrast, another subset of individuals with positive patch tests (group B) displayed attenuated molecular and histological responses. These findings suggest variability between individuals or different reactivity thresholds, which may be influenced by genetic factors, exposure time, or occupational activities (26). It is unclear whether the individual with a less intense response may develop later an accentuated response. Moreover, cytokines such as IL-9, IL-13, members of IL-20 family as IL-19, IL-20, and IL24 are upregulated in the MI response. These IL-20 family cytokines enhance innate defense mechanisms and tissue repair processes at epithelial surfaces, possibly contributing to the maintenance of homeostasis in the ACD reaction (27). This approach has not been previously described in ACD.

Histopathological alterations in high ACD responders to MI (ACD-A) included spongiosis, micro vesicle formation, and perivascular lymphohistiocytic infiltrates. These are classic features of ACD, characterized by lymphocyte, Langerhans cell, and macrophage infiltration in the upper dermis, indicating an active inflammatory process (28). Our previous findings with MCI/MI (Kathon CG) also identified type 2 macrophages in the perivascular skin (15). Indeed, macrophages can produce IL-19, IL-20, and IL-24 when activated by TLR ligands in other models of inflammation (29). In the present study, these cytokines were markedly elevated in group A, suggesting a contribution from both macrophages and T cells. These cytokines are known to signal through the STAT3 pathway in keratinocytes (30).

Interestingly, the comparison of upregulated DEGs in ACD-A MI revealed 1,160 genes when compared with the healthy control group, 1,588 genes when compared with group B, and 3,186 genes when compared with their own response to saline. These findings indicate that ACD-A MI, relative to the saline patch test, exhibited minimal DEG changes in the negative control and marked hyperreactivity to MI in the ACD-A group. As shown in the Venn analysis, exclusive DEGs were identified in ACD-A MI compared with the other groups, indicating specific molecular signatures associated with group differences, including the low-reactivity group B.

Remarkably, several cytokines were frequently upregulated in the ACD-A MI group, including IL-24, NTRK1, IL-9, IL-13, IL-18, IL-6, and CXCL8. Interestingly, in the enrichment analysis of Th2 cytokines, we observed upregulation of IL-13 but not IL-4. In a previous study on ACD induced by the MCI/MI compound (Kathon), we reported upregulation of IL-4 and minimal IL-13 expression (7). It remains unclear whether this difference between MCI/MI and MI is due to distinct kinetics of cytokine expression; it is possible that IL-4 peaked earlier than 48 hours in response to MI, but other time points were not assessed in this study. Furthermore, it is possible that the MI response may exhibit more epitopes than when associated with MCI. In addition, our analysis allowed us to add more comparison groups, such as ACD-A exposed with saline, as control group, emphasizing the presence of IL-13.

Among the cytokines associated with Th2 responses, IL-9 was upregulated in the ACD-A group, both at the transcriptomic level and in protein expression within the dermis. A variety of immune cells may contribute to IL-9 secretion, including Th9 cells, type 2 innate lymphoid cells (ILC2s), and mast cells. In ACD induced by nickel, IL-9 has been shown to play a regulatory role in Th1 responses by directly modulating Th1 cells and promoting IL-4 secretion (31). Notably, in our study, IFN-related genes were not identified as DEGs in any of the subgroups analyzed.

Moreover, we identified IL-24 as an upregulated DEG, along with increased protein expression in the dermis. IL-24 is secreted by several cell types in the skin, including CD4^+^ T cells, NK cells, mast cells, and keratinocytes. In the deconvolution analysis, we observed an increased proportion of CD4^+^ T cells and M2 macrophages, both of which may contribute to IL-24 production. The role of IL-24 in response to other allergens, such as para-phenylenediamine, has been correlated with clinical symptoms (32). In addition, IL-24 has been described as a novel player in the pathogenesis of allergic skin inflammation, including psoriasis, arthritis, inflammatory bowel disease, and atopic dermatitis (AD) (33). Since both IL-9 and IL-24 were identified as upregulated DEGs and showed increased protein expression in response to MI, further investigation of Th9 and IL24 involvement in these responses is warranted.

Interestingly, upregulation of NTRK1 was identified for the first time in ACD in response to MI. Sensory pathways involving NTRK1 may be exacerbated by scratching, a common feature of ACD. There is a direct relationship between IL-13 and the NTRK1 receptor, which binds to nerve growth factor (NGF), an early transcriptional target of IL-13 whose expression, is STAT6-dependent (34). In addition to its neurotrophic role, NGF also functions as an inflammatory mediator, contributing to sensations of pain and pruritus (35).

Several factors, including IL-6 and CXCL8, were markedly increased in the ACD-A MI group and may contribute to skin barrier disruption causing spongiosis, the release of damage-associated molecular patterns (DAMPs), and the activation of additional inflammatory pathways by damaged keratinocytes (36). These mediators can also be induced in irritant contact dermatitis (ICD) (30), which could potentially confound interpretation if they were also detected in controls or saline-treated samples. However, no such induction was observed in these groups, indicating a minimal immune response in the absence of allergen exposure.

In contrast, our results draw attention to the negative regulation of the genes IL-37, IL18, FLG2 and LORICRIN in ACD samples by MI. Although IL-18 is a proinflammatory factor, in some conditions it induces the Th1 response, which does not appear to be induced in this ACD reaction. IL-18, together with IL-12 and IL-15, plays an important role in the production of IFN-gamma by T cells, although it also induces Th2 cells (37). In addition, the reduction in IL-18 may be related to its rapid half-life of approximately 16 hours, which may contribute to the decrease in its local concentration (38). IL-37 belongs from the IL-1 family stands out for its regulatory role, produced mainly by monocytes, macrophages, dendritic cells, and epithelial cells in the skin and intestine in response to inflammation. It plays a key role in mitigating excessive inflammation and preventing tissue damage by regulating innate and adaptive immune responses. In fact, downregulation of IL-37 may favor the chronicity of memory effector T cells in ACD. Furthermore, the downregulation of FLG2 and LORICRIN—key components of tight junctions and the stratum corneum—has been observed in the skin of patients with severe ACD following PPD exposure, even in the absence of clinical symptoms (39). These findings indicate that skin barrier disruption induced by potent contact allergens and proinflammatory mediators may contribute to tissue damage and disease severity.

Transcriptomic data also revealed that, in individuals who were non-reactive to MI, there was no induction of genes associated with irritative responses. Overall, few transcriptomic studies have been conducted specifically on MI. Our findings demonstrate, at the molecular level, distinct differences between high and low responders to MI. Key factors involved in this response include IL-9 and, IL-24, which should be further investigated to better elucidate the pathogenesis of ACD induced by MI.

## Data Availability

The data presented in the study are deposited in the NCBI GEO repository, accession number GSE305246.

## Supplementary material

Supplementary Figure 1 Volcano plot of differentially expressed genes (DEGs) between ACD-A MI and ACD-A saline groups. Genes significantly upregulated in the ACD-A MI group are shown in red, downregulated genes are shown in blue, and non-significant genes are shown in grey. The x-axis represents the log_2_ fold change (log_2_FC), and the y-axis represents the –log_10_ adjusted p-value. Several immune-related and inflammatory genes, including IL6, IL13, IL20, IL9, CXCL1, and NTRK1, were among the most significantly upregulated.

Supplementary Figure 2 Relative expression of cytokine and neuroinflammatory genes in skin biopsies from individuals with methylisothiazolinone-induced allergic contact dermatitis (MI-ACD) and healthy controls (HC), measured by RT-qPCR. MI-ACD samples were divided into ACD-A (more reactive, red) and ACD-B (less reactive, green); HC are shown in black. Expression of IL-24, NTRK1, IL-9, IL-6, IL-13, IL-18, and CXCL8/IL-8 was normalized to endogenous controls using the 2^−ΔCt method. Data are mean ± SD. Kruskal-Wallis test revealed significant differences in NTRK1, IL-9, IL-6, and IL-18.
