# Supplementary figures and images for: IL-9 and IL-24 biomarkers in the transcriptional signature of contact dermatitis to methylisothiazolinone
Source: Front Immunol. 2025 Nov 28;16:1685396. doi: 10.3389/fimmu.2025.1685396 (PMC12698526; doi:10.3389/fimmu.2025.1685396)

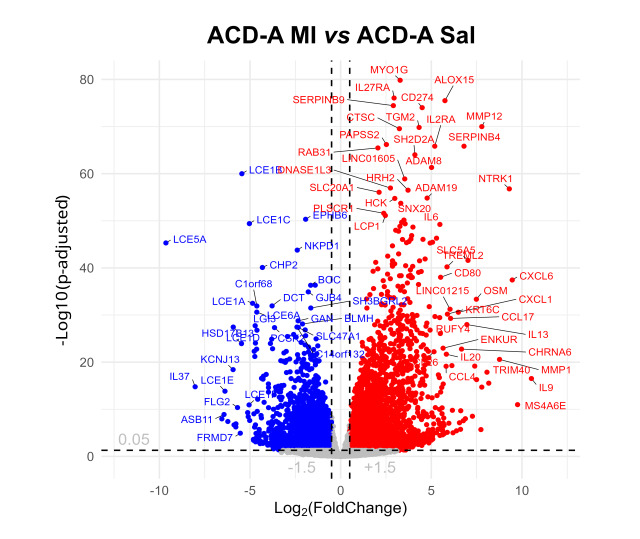

Supplement: Supplementary Figure 1 — Volcano plot of differentially expressed genes (DEGs) between ACD-A MI and ACD-A saline groups. Genes significantly upregulated in the ACD-A MI group are shown in red, downregulated genes are shown in blue, and non-significant genes are shown in grey. The x-axis represents the log2 fold change (log2FC), and the y-axis represents the –log10 adjusted p-value. Several immune-related and inflammatory genes, including IL6, IL13, IL20, IL9, CXCL1, and NTRK1, were among the most significantly upregulated. [file Image1.jpeg]

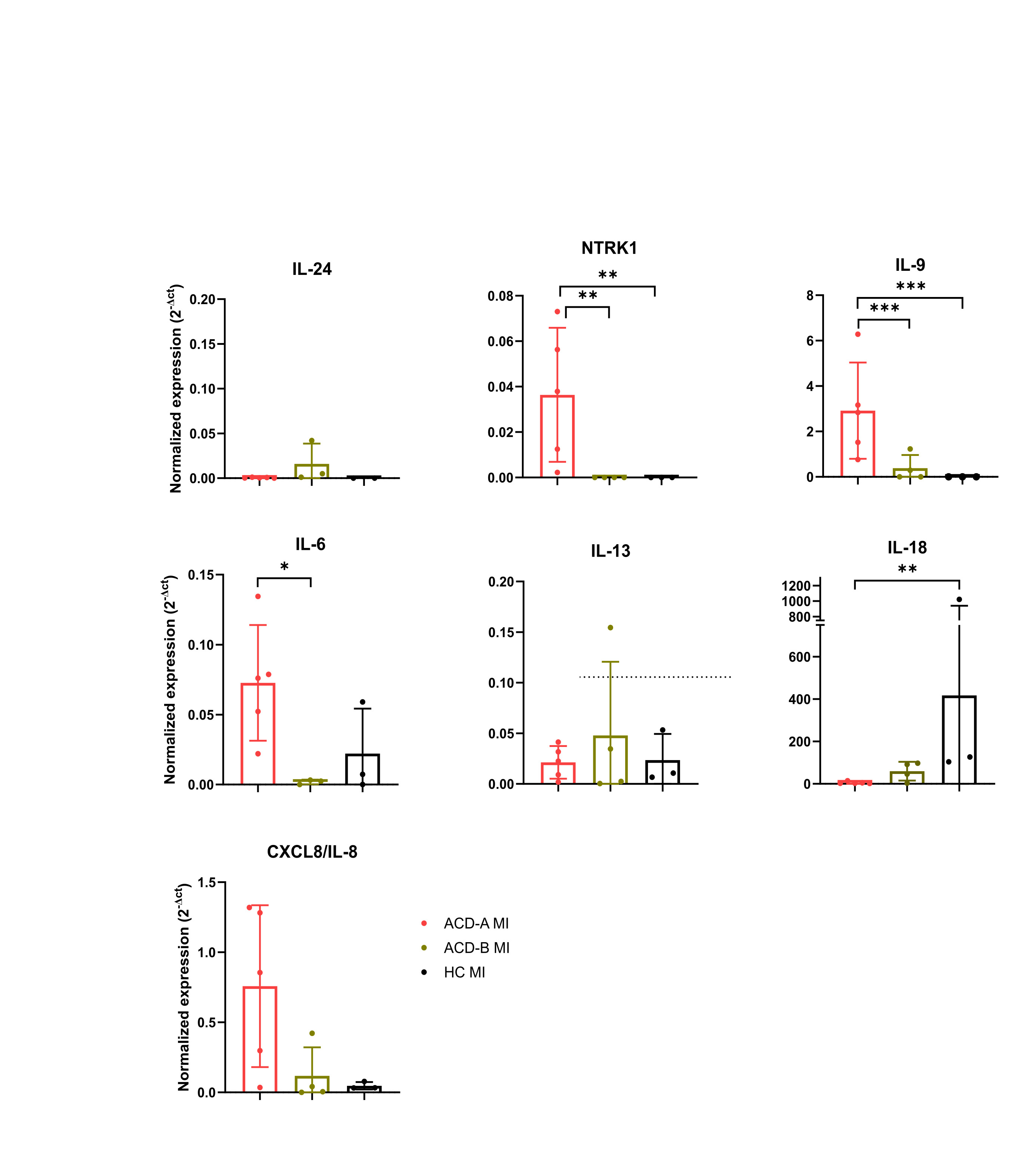

Supplement: Supplementary Figure 2 — Relative expression of cytokine and neuroinflammatory genes in skin biopsies from individuals with methylisothiazolinone-induced allergic contact dermatitis (MI-ACD) and healthy controls (HC), measured by RT-qPCR. MI-ACD samples were divided into ACD-A (more reactive, red) and ACD-B (less reactive, green); HC are shown in black. Expression of IL-24, NTRK1, IL-9, IL-6, IL-13, IL-18, and CXCL8/IL-8 was normalized to endogenous controls using the 2^−ΔCt method. Data are mean ± SD. Kruskal-Wallis test revealed significant differences in NTRK1, IL-9, IL-6, and IL-18. [file Image2.jpeg]
