# Supplementary material for: IL-9 and IL-24 biomarkers in the transcriptional signature of contact dermatitis to methylisothiazolinone
Source: Front Immunol. 2025 Nov 28;16:1685396. doi: 10.3389/fimmu.2025.1685396 (PMC12698526; doi:10.3389/fimmu.2025.1685396)
Supplement: Supplementary file 3 [file Table1.docx]

**Supplementary Table 1. Oligonucleotide sequences used in qPCR experiments.**
List of forward and reverse primer sequences employed for quantitative PCR (qPCR) analysis of target genes, including NTRK1, IL9, IL18, IL13, IL6, CXCL8/IL8, and the reference gene GAPDH. Primer sequences are presented in the 5′→3′ orientation.

| *Primer* | Sequência |
| --- | --- |
| NTRK1 Foward | 5’- AACAGCACATCTGGAGACCC – 3’ |
| NTRK1 Reverse | 5’- CAGCCGGGCGGTTGAT-3’ |
| IL-9 Foward | 5’- GGGATCGTGGACATCAACTTC-3’ |
| IL-9 Reverse | 5’-GAAGCATGGTCTGGTGCAGTT-3’ |
| IL-18 Foward | 5’-GACGCATGCCCTCAATCC-3’ |
| IL-18 Reverse | 5’-CTAGAGCGCAATGGTGCAATC-3’ |
| IL-13 Foward | 5’-GCAATGGCAGCATGGTATGG-3’ |
| IL-13 Reverse | 5’-CTGCACAGTACATGCCAGCT-3’ |
| Il-6 Foward | 5’- CCTGAGAAAGGAGACATGTAA-3’ |
| IL-6 Reverse | 5’GGCAAGTCTCCTCATTGAATCC-3’ |
| CXCL8/IL-8 Foward | 5’-TGTGTGTAAACATGACTTCCAAGCT-3’ |
| CXCL8/IL-8 Reverse | 5’-GCAAAACTGCACCTTCACACAG-3’ |
| GAPDH Foward | 5’- GAAGGTGAAGGTCGGAGT -3’ |
| GAPDH Reverse | 5’- GAAGATGGTGATGGGATTTC -3’ |
